# Collecting verbal autopsies: improving and streamlining data collection processes using electronic tablets

**DOI:** 10.1186/s12963-018-0161-9

**Published:** 2018-02-01

**Authors:** Abraham D. Flaxman, Andrea Stewart, Jonathan C. Joseph, Nurul Alam, Sayed Saidul Alam, Hafizur Chowdhury, Meghan D. Mooney, Rasika Rampatige, Hazel Remolador, Diozele Sanvictores, Peter T. Serina, Peter Kim Streatfield, Veronica Tallo, Christopher J. L. Murray, Bernardo Hernandez, Alan D. Lopez, Ian Douglas Riley

**Affiliations:** 1 Institute for Health Metrics and Evaluation, University of Washington, Seattle, WA USA; 2 International Centre for Diarrhoeal Disease Research, Dhaka, Bangladesh; 3 School of Population and Global Health, University of Melbourne, Parkville, VIC Australia; 4 Research Institute for Tropical Medicine, Muntinlupa City, Philippines

## Abstract

**Background:**

There is increasing interest in using verbal autopsy to produce nationally representative population-level estimates of causes of death. However, the burden of processing a large quantity of surveys collected with paper and pencil has been a barrier to scaling up verbal autopsy surveillance. Direct electronic data capture has been used in other large-scale surveys and can be used in verbal autopsy as well, to reduce time and cost of going from collected data to actionable information.

**Methods:**

We collected verbal autopsy interviews using paper and pencil and using electronic tablets at two sites, and measured the cost and time required to process the surveys for analysis. From these cost and time data, we extrapolated costs associated with conducting large-scale surveillance with verbal autopsy.

**Results:**

We found that the median time between data collection and data entry for surveys collected on paper and pencil was approximately 3 months. For surveys collected on electronic tablets, this was less than 2 days. For small-scale surveys, we found that the upfront costs of purchasing electronic tablets was the primary cost and resulted in a higher total cost. For large-scale surveys, the costs associated with data entry exceeded the cost of the tablets, so electronic data capture provides both a quicker and cheaper method of data collection.

**Conclusions:**

As countries increase verbal autopsy surveillance, it is important to consider the best way to design sustainable systems for data collection. Electronic data capture has the potential to greatly reduce the time and costs associated with data collection. For long-term, large-scale surveillance required by national vital statistical systems, electronic data capture reduces costs and allows data to be available sooner.

## Background

Accurate and timely data on the cause of death (COD) distribution within a population forms a key component of a functioning health information system [1]. These data are crucial for informing discussions of health policy and priority setting [2]. However, most countries do not gather any cause of death data, or they gather data that is incomplete or inaccurate [3]. Over the last few decades, modest progress has been made to increase the number of deaths registered in civil registrations systems and to increase the quality of medical certification of the cause of death [4]. It is now recognized that complementary methods of data collection, such as verbal autopsy (VA), are required to provide much needed COD data for deaths that are not medically attended and to act as a stepping stone for the development of fully functioning health information systems [5]. A number of countries, such as India [6], Bangladesh [7], Brazil [8], Sri Lanka [9], China [10], and Tanzania [11], have already incorporated verbal autopsy into their routine health surveillance systems and the World Health Organization (WHO) has called for its greater used to monitor the levels and trends of causes of death within populations [12].

There have traditionally been three major obstacles preventing widespread adoption of verbal autopsy for generating population level estimates of COD. The first obstacle involves creating an interview instrument that can be completed in a reasonably short amount of time, yet is able to accurately distinguish between multiple causes of death. An empirically designed and validated survey instrument now exists [13].

The second obstacle has been a reliance on physician certification of verbal autopsies to ascertain the cause of death. This is both expensive and time consuming. Additionally, in many of these settings, there is a shortage of doctors, and requiring physicians to spend their time certifying VA interviews reduces the time that they can practice clinically. Recent advances in algorithms for automatic computer coding of verbal autopsies have been shown to be more accurate, consistent, and faster [14, 15]. Thus this burden can be avoided while simultaneously providing higher quality estimates.

The final major obstacle involves the time and costs associated with entering paper versions of individual interviews into a computer database. Providing accurately aggregated data in a timely manner is currently a bottleneck in most settings and must be overcome in order to scale up use of verbal autopsy.

Electronic data capture systems now provides a feasible alternative to the traditional paper-and-pencil approach for collecting surveillance data [16]. These systems provide three key advantages. First, well-designed survey instruments have been shown to eliminate data collection errors related to missingness in required fields and questionnaire skip logic [17, 18]. Second, capturing data electronically at the point of contact removes the need for the subsequent data entry, which reduces the time required to collect and aggregate results [18, 19]. Third, the costs of paper printing and data entry are often greater than the upfront cost of purchasing electronic devices [18–21]. These findings support the conclusion that electronic data capture systems are practical and cost-effective in low-resource settings.

In this paper, we add to this body of knowledge in two key ways. First, we quantify the difference in time required to enter and aggregate information from verbal autopsy interviews (VAIs). Second, we begin to explore the tradeoff between cost and time for data entry of VAIs. This information is crucial for designing cost-effective VA programs for routine surveillance.

## Methods

### Data collection process

**Fig. 1:**
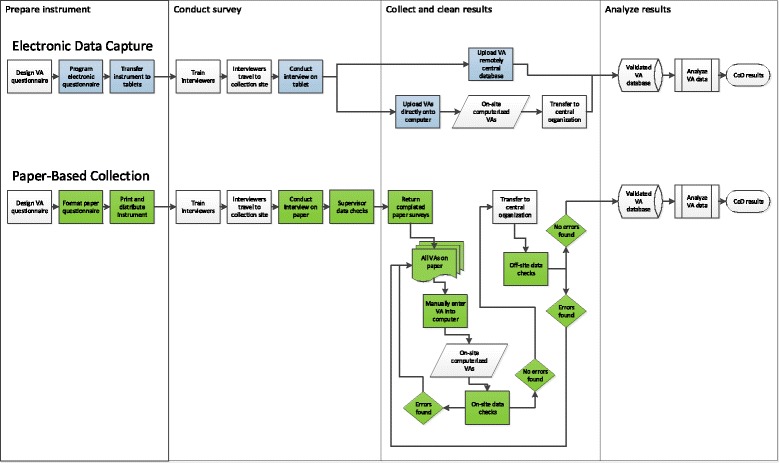
Flowchart of process for collecting VAIs comparing Electronic Data Capture with Paper-Based Collection

The process of collecting VAIs with pencil and paper or with electronic tablets shares some key steps (Fig. 1). In both cases, a VA questionnaire has to be designed and translated into the language and vocabulary of the survey population [22]. Interviewers must be trained and travel to the collections sites [23]. Afterwards, the collected VAIs must be entered into a centralized database [12]. Once centralized, this database is analyzed to produce individual cause of death diagnoses and population level estimates of cause specific mortality fractions [24].

The two processes also share key steps which are implemented slightly differently [12]. First, copies of the questionnaire must be created and distributed. For VAIs collected on paper, this involves printing paper copies. For collection on electronic tablets, this involves programing a questionnaire, procuring suitable tablet devices and downloading the survey instrument to the device. The second similar step involves conducting the interview either on paper or on a tablet [12]. The third shared step involves centralizing the data. For paper surveys, results must be manually entered into a computer. For surveys collected on tablets, VAIs are uploaded into a central database [25].

When surveys are collected on paper, there is an additional iterative set of steps involved in validating the data. First, during data collection, supervisors must check surveys as they are completed. The surveys are then entered into a results database on the computer. The computerized data must be checked again to ensure that the computer record exactly matches the paper survey. Next, the computerized data must be checked for completeness and accuracy. This involves validating the essential data fields and checking for logical inconsistencies [26]. For surveys collected on electronic tablets, all of these steps are automated. The survey program enforces entry of required data fields before proceeding and includes skip logic to prevent logical inconsistencies. Also, since data are electronically transferred, there is nothing to be physically shipped by supervisors to a central location.

This study focuses on assessing the differences in the administrative burden between collecting VAIs with electronic tablets vs. with pencil and paper. Both methods share a common starting point and end point. Namely, the starting point is a well-designed questionnaire, such as the Population Health Metrics Research Consortium (PHMRC) Shortened Questionnaire [27, 13]. The end point is a cleaned digital dataset of responses to VAI interview questions, which has been checked for missing data and internal consistency. From this dataset, additional analysis is always performed, such as estimating the leading causes of death, but this subsequent analysis does not depend on the method by which the VAIs are collected and therefore its cost and time are not considered in the present paper [14].

### Sites and instruments

In this study, we collected and analyzed data about administering VA studies on paper and electronic instruments at the same two sites. Paper-based collection was conducted using the PHMRC Full Questionnaire. This instrument is described in detail elsewhere [27]. In summary, the instrument contains a five-page general section of closed-response questions and an age-specific section ranging from 12 to 15 pages of closed-response questions. The survey includes 127 to 183 question depending on the age of the decedent, but not all questions are answered due to skip logic. In addition, the survey includes a single page for transcribing an open-ended narrative. Tablet-based electronic collection was conducted using the PHMRC Shortened Questionnaire. This instrument and how it was constructed is also described in detail elsewhere [13]. In summary, the questions from the PHMRC Full Questionnaire that contained the least information value for predicting cause of death were dropped, resulting in a total of 67 to 109 closed-ended questions depending on the age of the decedent. Additionally, instead of transcribing the entire open narrative verbatim, interviewers recorded if a set of informative keywords were mentioned during the narrative. The electronic instrument was created using Open Data Kit (ODK) software, an open-source tool for developing mobile data collection forms and streamlining the aggregation of data on a server [25]. VAIs were collected using Samsung Galaxy Tab 2 tablets.

VAIs were collected on paper in Matlab, Bangladesh between January 2011 and May 2012, and in Bohol, Philippines between November 2010 and December 2012. VAIs were collected from the same sites on electronic tablets from December 2012 to July 2013.

### Analysis

To assess the differences in the administrative burden of collecting VAIs on paper and on electronic tablets, site coordinators recorded the cost and time required to complete different steps of the collection process. Data were collected on the cost of printing paper surveys, the cost of procuring electronic tablets, the cost of personnel and computers for data entry, the time spent training interviewers to use each instrument, and the time spent entering and checking data.

**Fig. 2:**
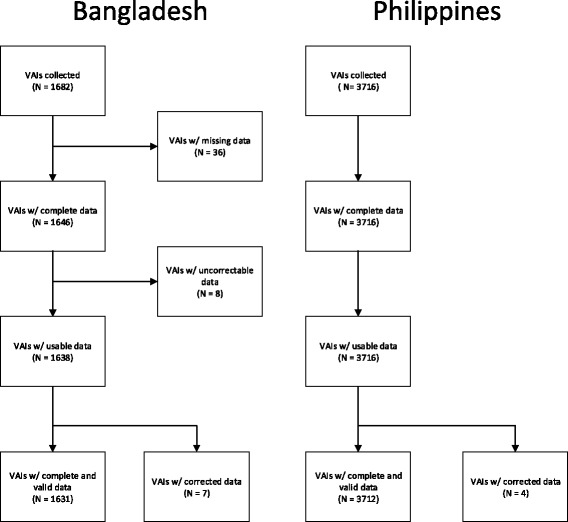
CONSORT diagram showing how errors in pencil-and-paper collected VAIs limit data usability

For both sites, the date of the interview was recorded. For surveys collected on paper, the date the interview was checked by a supervisor and the date of data entry was also recorded. Some dates had data entry errors that resulted in implausible date sequences. These records were individually examined and compared with the range of dates from all records. In most cases, it was obvious that the year in one of the recorded fields had been entered incorrectly. This was most common when the interview was conducted in one calendar year and either the check or the entry occurred in the next calendar year. Incorrect dates were manually corrected before calculating time intervals (Fig. 2).

From these data, we estimated the difference in cost required to field verbal autopsy surveillance using paper and pencil versus using electronic tablets. To estimate the material cost of paper and pencil surveys we assumed that the cost of printing was proportional to the number of VAIs collected. The cost of data entry varies with the amount of time allowed for data entry. We estimated the cost of data entry with the constraint that all the data must be entered within three, six, or 12 months. We also assumed that VAIs must be double entered for data quality reasons. The average number of VAIs entered per month was calculated from the recorded dates of entry and was assumed to be constant. Thus, the number of enterers needed varied both due to the time constraint and the number of VAIs collected. The cost for data entry for a single enterer was calculated as the one-time cost of a computer plus the monthly salary multiplied by the number of months required. This was multiplied by the number of enterers require. To estimate the material cost of tablets, the cost of the tablet was multiplied by the number of tablets required. Estimates were generated for 20 to 500 tablets. Since there is no data entry, this was the only cost associated with tablets. Estimates were generated for up to 30,000 VAIs. Data were not collected for the salaries of the field teams or other operational cost. These were assumed to be the same whether the interviewers used electronic tablets or paper and pencil. The model used to estimate cost was

$$ {\mathrm{Cost}}_{\mathrm{tablets}}={\mathrm{Price}}_{\mathrm{Tablet}}\times {N}_{\mathrm{Tablet}\mathrm{s}}; $$

$$ {\mathrm{Cost}}_{\mathrm{paper}}={\mathrm{Price}}_{\mathrm{Printing}}\times {N}_{\mathrm{VAI}}+\left({\mathrm{Price}}_{\mathrm{computer}}+{\mathrm{Price}}_{\mathrm{enterer}}\times {\mathrm{Time}}_{\mathrm{worked}}\right)\times \mathrm{Enterers}, $$

where

$$ \mathrm{Enterers}=2\times \left\lceil \frac{N_{\mathrm{VAI}}}{{\mathrm{Rate}}_{\mathrm{entry}}\times {\mathrm{Time}}_{\mathrm{allotted}}}\right\rceil, $$

Cost_tablets_ is the total cost of using tablets, Price_Tablet_ is the cost of a single tablet, N_Tablets_ is the number of tablets needed, Cost_paper_ is the total cost of using paper and pencil, Price_Printing_ is the price of printing a single VAI questionnaire, N_VAI_ is the number of VAIs collected, Price_computer_ is the price of a single computer used for data entry, Price_enterer_ is the monthly salary of a single enterer, and Time_worked_ is the length of time needed to enter the all the VAIs. Rate_entry_ is the number of VAIs a single enter can enter in a month and Time_allotted_ is the amount of time allowed for data entry. The number of enterers needed is always rounded up to the next whole number and is multiplied by two because data are double entered by independent enterers.

## Results

This study includes a total of 5398 VAIs collected on paper and 516 VAIs collected on electronic tablets. In Bangladesh, 1682 were collected on paper and 316 were collected on tablets. Of the VAIs collected on paper 1417 (84%) were adults, 129 (8%) were children, and 136 (8%) were neonates. The sample of VAIs collected on tablets in Bangladesh included 246 (78%) adults, 30 (9%) children, and 40 (13%) neonates. In the Philippines, 3716 were collected on paper and 200 were collected on tablets. Of the VAIs collected on paper 2945 (79%) were adults, 258 (7%) were children and 513 (14%) were neonates. The sample of VAIs collected on tablets in the Philippines included 120 (60%) adults, 40 (20%) children and 40 (20%) neonates.

In Bangladesh questionnaires were printed at a cost of 20 Taka per survey and electronic tablets cost 32,000 Taka. This is a unit cost of $0.257 (all dollar amounts in 2013 US dollars) per VAI for paper and $393.80 per interviewer for tablets. For data entry in Bangladesh, 80,000 Taka ($984.49) was spent on a computer and $6000 was spent on personnel to enter the data over 15.5 months giving a monthly salary of $387.10. In the Philippines, $115 per month was spent for 25 months and 15,000 pesos was spent for each tablets. This is a unit cost of $0.774 per VAI for paper and $365.76 per interviewer for tablets. For data entry in the Philippines, $1000 was spent on a computer and $326 per month was spent on personnel to enter the data. Table 1 summarizes the unit cost of materials, personnel and computer hardware at both sites.

**Table 1:** Unit costs and rates of parameters used to estimate costs for paper- and tablet-based VAIs surveys in Bangladesh and the Philippines

	Bangladesh	Philippines
Printing cost per paper VAI	$0.246	$0.774
Cost of a single electronic tablet	$393.78	$365.76
Cost of a computer for data entry	$984.49	$1000.00
Monthly salary of data enterer	$384.62	$326.00
Average number of VAI per person per month	107.8	145.7

*Note: All prices in 2013 US dollars

The amount of time needed to train interviewer to use the paper instrument was 6 days in Bangladesh and 5 days in Philippines. For the tablet-based survey, both sites used interviewers who had previously been training in how to use the paper version. In Bangladesh, there were 2 days of training on the tablets. In the Philippines, there was 1 day of training.

In Bangladesh, for paper forms, the amount of time from interview to data entry ranged from one to 419 days, with a median of 98 days. It took between zero and 405 (median 49) days for the survey to be checked by a supervisor. After it was checked, it took between zero and 248 (median 23) days to be entered into a computer. In the Philippines, the time ranged from four to 243 days with a median of 83 days. It took between zero and 151 (median 59) days for the survey to be checked by a supervisor and between zero and 196 (median 21) days to be entered into a computer. For VAIs collected on tablets, data was uploaded to the central database or transferred to a supervisor’s computer in a maximum of 1 day for Bangladesh and 2 days for the Philippines. Table 2 summarizes the amount of time spent on training interviewers, checking data, and entering data.

**Table 2:** Time comparison for paper- and tablet-based VAIs in Bangladesh and the Philippines

	Bangladesh		Philippines	
	Paper (*n* = 1682) median (min, max)	Tablet (*n* = 316)	Paper (*n* = 3716) median (min, max)	Tablet (*n* = 200)
Time for interviewer training	6 days	2 days^a^	5 days	1 day^a^
Time between interview and check by supervisor	49 days	N/A	59 days	N/A
	(0, 405)		(0, 151)	
Time between check by supervisor and data entry	23 days	N/A	21 days	N/A
	(0, 248)		(0, 196)	
Time between interview and data entry	98 days	1 day	83 days	2 days
	(1, 419)		(4, 243)	

^a^Note: Data collectors were previously trained on paper VA instruments

In paper forms, 19 records had logically impossible dates, 15 in the Bangladesh dataset and four in the Philippines. An additional 36 records in the Bangladesh dataset did not list a supervisor check date. We were able to correct seven of the records in the Bangladesh dataset and all four of the records in the Philippines dataset. Of the remaining eight records in the Bangladesh dataset two had supervisor check dates which occurred before the listed interview date and six had supervisor check dates which occurred after the data had been entered. For all eight of these records the dates of the interview and data entry were consistent and plausible. These records were included in calculating to the total time between interview and entry date, but excluded from calculating times involving supervisor check dates. Figure 2 describes which observations were used to calculate times.

**Fig. 3:**
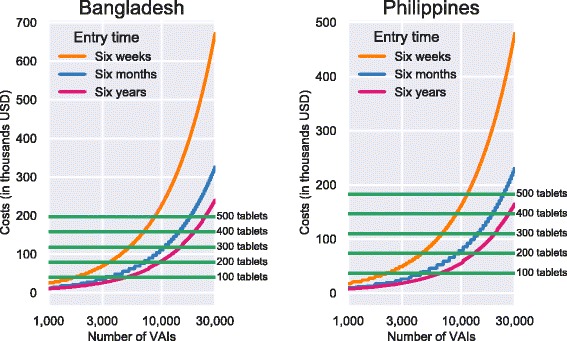
Estimated cost for collecting a given number of VAIs (on a log scale) using different number of tablets or time constraint for data entry for Bangladesh and Philippines

Figure 3 shows the results of the cost model using the parameters in Table 1 and varying the number of tablets needed and the amount of time allotted for data entry. The cost of collecting data on electronic tablets does not depend on the number of VAIs collected. The jaggedness in the line showing the cost of data entry within 6 months comes from the cost of purchasing additional computers for data entry so that all enterers can work simultaneously. To enter 10,000 VAIs in 6 months at the rate of data entry in Bangladesh it would take 32 enterers. To enter 10,000 VAIs in 6 months at the rate of data entry in the Philippines it would take 24 enterers. If 10,000 VAIs were collected in Bangladesh and the data needed to be entered in 6 weeks, it would be cheaper to buy 500 electronic tablets instead of paying for 124 data enterers and computers to enter data. In the Philippines, it would be cheaper to buy 400 electronic tablets instead of 92 data enterers. If data did not need to be entered for 6 years and 10,000 VAIs were collected, in Bangladesh 200 tablets could be purchased for approximately the same cost as entering the data. In the Philippines, 150 tablets could be purchased for approximately the same cost as entering the data over 6 years.

## Discussion

This study shows, for the first time, the savings in time and the large potential savings in cost of using electronic tablets for verbal autopsy interviews, especially when collecting a large number of VAIs. We found that individual records collected on tablets were available for analysis around 3 months earlier than pencil and paper data collection and the entire dataset was available over 8 months earlier. With electronic tablets, the costs are mainly are the upfront cost of the tablets, whereas with paper-and-pencil surveys the bulk of the costs are for data entry. More studies are now using electronic data collection to collect survey data in the field [28, 29]. This includes household surveys in low- and middle-income countries [21, 30, 19, 20, 31].

The result of our cost model agree with previous studies. For small-scale surveys, the cost of hardware for electronic data capture is higher than the cost of printing and data entry, which yields a higher total cost [14]. However, as survey size increase, the cost of data entry grows and electronic data capture can become cheaper overall [16, 20, 18, 32]. For projects that require fewer than 150 tablets and collect over 10,000 surveys, the upfront cost of the tablets will likely be substantially less than the cost of data entry. If the data need to be entered in a shorter timeframe, the cost of paying more data enterers quickly exceeds the cost of direct data capture. These time estimates are only for the data entry, not the cause of death analysis. If analysis is expected to take additional months, or perhaps years, the estimates will not be available for even longer. This concern is avoided for systems in which the electronic data can feed directly into software that codes the cause of death, further streamlining the process.

In this study, we also demonstrate that electronic data capture greatly reduces the time between data collection and analysis. Timely population estimates of COD are an essential input to health information systems. Out of date information may be less useful for policy decisions or, worse, may be obsolete and lead to poor decisions. These estimates should rely on as timely data as is available. For VAIs collected on paper and pencil, the full dataset is not available to be analyzed for six to 14 months after it is collected. For electronically collected VAIs, the full dataset can be analyzed just a few days after data collection has finished. If the analysis reveals issues which require follow-up, it is likely the original data-collectors are still in the area. If these issues are found months later, it may be very costly to return to the site to clarify the issue. The time lag between data collection and analysis is an important consideration for designing effective health information systems. Theoretically, one person could eventually enter 30,000 VAIs into a database, which would save the expenses related to purchase and maintaining multiple computers for data entry, but the unentered, collected data that cannot be accessed is essentially worthless.

Electronic data capture also shows the potential to reduce data error. Well-designed electronic instruments can effectively eliminate user error by preventing continuation or completion of the survey until all fields are appropriately filled out. Our results show no skip logic or invalid entry errors for VAIs collected on electronic tablets. Other research utilizing electronic instruments designed to eliminate entry error have achieved the same result [17, 18]. Other studies have found that rate entry errors is similar or lower than paper-based surveys, even when the interviewers are not very familiar with technology [19, 33, 34]. Our study did not collect information to compare the reliability or rates of measurement errors between the two collection methods. Currently, there are very little baseline data for comparison, but as verbal autopsy collection scales up, it will be important to measure the accuracy of each data collection method.

The instruments we used included an open narrative section. For the interviews collected on tablets, we only collected information on whether the respondent mentioned predetermined keywords, instead of transcribing the whole narrative verbatim. This reduces the burden during data collection, especially for interviewer who are not used to transcribing large amounts of text on an electronic keyboard, but also reduces the data entry workload. Additionally, interviewers are able to listen for keywords in the language of the interview and mark the translated keyword. This further reduces work required to enter data. The open response has been found to improve predictive accuracy of cause of death algorithm [14]. It is useful to try to capture this information in a streamlined way that does not place excessive burden on data collection. We expect that processes using alternative instruments for collecting VA on tablets which use this “checklist” approach for capturing the narrative section of the interview, such as the WHO 2016 Verbal Autopsy Questionnaire, [35] would have similar benefits in time and cost when compared to paper.

The use of electronic tablets for data collection also have a number of indirect effects worth considering. Large-scale data collection operations often employ local community members. This can provide economic opportunity and foster good community relations. In subsequent pilot studies involving collecting verbal autopsy, we have found that participants welcome the use of tablets over paper and pencil and see their inclusion as a more formal interaction with the government. The tablets for this study were designated to be used only for this data collection effort, so we locked all other apps related to entertainment and social media to help protect the confidentiality of the collected data, although conceivably, the tablets could have been used for similar data collection projects. We did not attempt to examine indirect effects of other potential uses of electronic tablets in this study. Lastly, shifting human capacity from paper and pencil clerical work to tablet setup and maintenance and training interviewers to use electronic tablets could help build local technical capacity, which is something to study in the future.

This study did not comprehensively capture the time and cost associated with collecting VAIs. We did not assess the time and costs associated with constructing an instrument suitably tailored to the target population. Other studies have reported on the complexity and time required to develop [16] and modify [36] electronic instruments. We also did not capture information about the initial time and cost required to configure tablets for use and install all the required software. These considerations are important for sporadic studies or for transitioning to electronic instruments, but may be less important for long-term surveillance with the same instrument. Also, this study was not able to compare the time required to train interviewers on just the tablets without an initial background in the survey itself. Previous work has shown that interviewer’s lack of familiarity with electronic devices before the study increase the time needed to complete an interview and increase error rates [19].

To fully assess the cost of fielding a study with electronic tablets, other operational costs must be quantified. Both the sites in this study had fairly reliable access to internet connections and were able to upload data to the central server frequently. In locations without consistent internet access it may be necessary to purchase memory cards for the tablets to back up data locally until reaching a location with internet access [21]. Our model assumes that the field teams are able to automatically upload data to a central server free of charge when they have internet access. In this study, this was accomplished with the open-source ODK Aggregate software and resulted in no additional cost to centralizing the data. Another important consideration is power supply, especially in rural settings without reliable access to electricity. This may require purchasing external batteries, solar chargers, adapters for charging in vehicles, or paying fees for charging stations [16, 19]. Other operational costs include replacing tablets, memory cards or chargers that are lost, damaged, or stolen [37]. For this study, we only collected a few hundred interviews in a restricted geographic area over a short time frame, so we were not able to adequately estimate costs associated with maintenance and wear-and-tear on tablet over the lifetime of their use. Likewise, there were cost associated with paper-based surveys that we were not able to quantify. These include the storage space for paper forms and office space and electricity for the computers used for data entry.

Another important limitation of this study is that we only estimated the cost of data entry using one VA instrument, the full length PHMRC Questionnaire. The time needed to enter a VAI depends on the complexity and length of the survey instrument. Additional time may also be required if an open narrative section with lengthy free text if collected. When we conducted the survey on electronic tablets we used a shortened questionnaire. The cost of data collection on electronic tablets would be the same regardless of whether we used the full or shortened instrument, since there is no cost associated with data entry. However, if we had used the shortened instrument with paper-based collection we would expect less time needed to enter VAIs and a higher entry rate. Our model estimates the cost of printing and entering the full-length PHMRC instrument. It is likely this is a small overestimate of the cost of using the paper version of the PHMRC Shortened Questionnaire.

## Conclusion

Most of the previous research has focused on the costs and accuracy of data collected on electronic devices. Our model shows that for large-scale verbal autopsy surveys, the cost of electronic data systems is less than paper-based systems. In this study, we show that the amount of time between data collection and analysis is also an important consideration. Verbal autopsy surveys conducted on tablets are available for analysis much sooner and provide data that are more accurate. This is essential for providing timely data for health policy and priority setting.

## Abbreviations

COD: Cause of death
ODK: Open data Kit
PHMRC: Population health metrics research consortium
VA: Verbal autopsy
VAI: Verbal autopsy interview
WHO: World Health Organization
